# Comparative Evaluation of the LAMP Assay and PCR-Based Assays for the Rapid Detection of *Alternaria solani*

**DOI:** 10.3389/fmicb.2018.02089

**Published:** 2018-09-03

**Authors:** Mehran Khan, Rongbo Wang, Benjin Li, Peiqing Liu, Qiyong Weng, Qinghe Chen

**Affiliations:** ^1^Fujian Key Laboratory for Monitoring and Integrated Management of Crop Pests, Institute of Plant Protection, Fujian Academy of Agricultural Sciences, Fuzhou, China; ^2^State Key Laboratory of Ecological Pest Control for Fujian and Taiwan Crops, Fujian Agriculture and Forestry University, Fuzhou, China

**Keywords:** *Alternaria solani*, early blight, *histidine kinase* gene, LAMP, real-time PCR, sensitivity, specificity

## Abstract

Early blight (EB), caused by the pathogen *Alternaria solani*, is a major threat to global potato and tomato production. Early and accurate diagnosis of this disease is therefore important. In this study, we conducted a loop-mediated isothermal amplification (LAMP) assay, as well as conventional polymerase chain reaction (PCR), nested PCR, and quantitative real-time PCR (RT-qPCR) assays to determine which of these techniques was less time consuming, more sensitive, and more accurate. We based our assays on sequence-characterized amplified regions of the *histidine kinase* gene with an accession number (FJ424058). The LAMP assay provided more rapid and accurate results, amplifying the target pathogen in less than 60 min at 63°C, with 10-fold greater sensitivity than conventional PCR. Nested PCR was 100-fold more sensitive than the LAMP assay and 1000-fold more sensitive than conventional PCR. qPCR was the most sensitive among the assays evaluated, being 10-fold more sensitive than nested PCR for the least detectable genomic DNA concentration (100 fg). The LAMP assay was more sensitive than conventional PCR, but less sensitive than nested PCR and qPCR; however, it was simpler and faster than the other assays evaluated. Despite of the sensitivity, LAMP assay provided higher specificity than qPCR. The LAMP assay amplified *A. solani* artificially, allowing us to detect naturally infect young potato leaves, which produced early symptoms of EB. The LAMP assay also achieved positive amplification using diluted pure *A. solani* culture instead of genomic DNA. Hence, this technique has greater potential for developing quick and sensitive visual detection methods than do other conventional PCR strategies for detecting *A. solani* in infected plants and culture, permitting early prediction of disease and reducing the risk of epidemics.

## Introduction

*Alternaria solani* (Ellis and Martin), a pathogen causing early blight (EB), is a major threat to global potato and tomato production (Song et al., 2011). EB produces symptoms on leaves, stems, petioles, twigs, and fruits, ultimately leading to defoliation, twig drying, and premature fruit fall, which can result in 35–78% fruit yield losses (Datar and Mayee, 1982; Grigolli et al., 2011). Control measures such as prophylactic methods, fungicide application, and the use of relatively resistant tomato cultivars are currently used to counter EB. At high disease densities, these procedures cannot entirely cope with EB (Rao et al., 2008); therefore, the development of rapid, sensitive, and reliable methods to assess pathogen populations or pathogen loads is crucial for timely and effective disease management. At present, most assays used in *A. solani* detection depend on visual assessment of the symptoms, including lesion diameter measurement and spore load count (Bock et al., 2010). These methods are time consuming and often cannot distinguish fine-scale differences in inoculum loads and plant resistant levels. They are also unsuitable for the evaluation of fungal development in the early phases of infection, before macroscopic symptoms are evident or sporulation occurs.

Pathogens involved in EB diseases include *A. solani* and *A. alternata*. Numerous studies have shown that these species can be isolated simultaneously from plants exhibiting typical EB symptoms (Leiminger, 2009; Latorse et al., 2010). To date, *Alternaria* species identification has relied on agar plate methods, through macroscopic examination of spore morphology. Likewise, disease scoring based on visually assessed symptoms cannot differentiate among diverse pathogens. Although different methods exist for the detection of *Alternaria* species in potato, these techniques do not permit quantification of the fungi *in planta*. Recently genome assembly of *A. solani* of potato and tomato is proposed, which might be very helpful to find out specific regions of the target pathogen (Wolters et al., 2018). The technique used most commonly to identify *Alternaria* species is conventional polymerase chain reaction (PCR) with species-specific primers based on the ribosomal DNA internal transcribed spacer. Real-time quantitative PCR (RT-qPCR) is a highly sensitive technique for the detection and quantification of specific nucleic acids (Taylor et al., 2001). In the current study, we describe multiple PCR-based assays used to assess the extent of *Alternaria* colonization in potato leaves during early disease development and to distinguish *Alternaria* species. These assays enable tracking of the advancement of *Alternaria* species in the host, which may contribute to a better understanding of EB epidemiology, and may be applicable in epidemiological investigations and disease management strategies.

PCR assays in current use have limited overall adaptability and applicability in disease identification (Ling et al., 2010); their drawbacks include the need for deluxe laboratory equipment and reagents, as well as proper training and technical expertise, which are often inaccessible in poorly resourced laboratory settings (Francois et al., 2011; Duan et al., 2014; Moradi et al., 2014). In previous studies, advantages and disadvantages of PCR assays and loop-mediated isothermal amplification (LAMP) have been described (Lau and Botella, 2017). LAMP is a novel technique that can effectively address the deficiencies of PCR-based methods (Notomi et al., 2000; Mori et al., 2013; Niessen, 2015).

The LAMP assay is a single-step procedure that requires four to six primers that bind laterally to distinct sites using strand-displacement *Bst* DNA polymerase, permitting extremely specific amplification under isothermal conditions (Notomi et al., 2000; Tomita et al., 2008; Li and Macdonald, 2015). The amplified products can be detected by gel electrophoresis, turbidimeter, lateral flow dipstick, or the naked eye (Mori et al., 2004; Mao et al., 2012; Niu et al., 2012; Meng et al., 2017); thus, LAMP can be applied in the field using small portable instruments, as well as in laboratories.

LAMP approaches have shown tolerance to inhibitory substances present in biological samples; their simple and rapid extraction methods allow users to avoid complicated DNA refinement protocols (Niessen, 2015; Khan et al., 2017; Si Ammour et al., 2017). LAMP is therefore a straightforward, sensitive, rapid, and cost-effective method that can be used for early diagnosis and *in-situ* testing of crop pathogens.

The objective of the current study was to compare the efficiencies of different PCR-based assays with the LAMP method to determine which was most suitable for rapid on-site diagnosis of *A. solani* based on the *histidine kinase* gene (HK1).

## Materials and Methods

### Fungal Culture Isolation and DNA Extraction

Isolates of *A. solani*, other *Alternaria* species, and oomycete fungi used in this study were sampled from different geographic counties of China and collected by the China General Microbiological Culture Collection Center; they are listed in **Table 1**. All isolates were cultured on potato dextrose agar (PDA) at an incubation temperature of 28°C for 7 days. Mycelia were harvested from the Petri dishes with a sterile scalpel, transferred to a 1.5-mL Eppendorf tube, and then air dried for 48 h and crushed with a grinder. DNA samples were extracted from the air-cooled mycelia using the Bioteke kit (Bioteke, Dalian, China) and quantified using a NanoDrop 2000 device (Thermo Fisher Scientific, Waltham, MA, United States); aliquots were diluted with RNA-free double-distilled water to 100 ng/μL and stored at -20°C until further use.

**Table 1:** Fungal isolates used in this study.

Species^a^	Host	No. of isolates	Source^b^	Conventional PCR	LAMP^c^	Real time-qPCR
*Alternaria solani*	*Solanum tuberosum*	8	Gansu, China	+	+	+
*Alternaria solani*	*Solanum tuberosum*	4	Hebei, China	+	+	+
*Alternaria solani*	*Solanum tuberosum*	6	Jiangsu, China	+	+	+
*Alternaria solani*	*Solanum tuberosum*	3	Ningde, China	+	+	+
*Alternaria solani*	*Solanum tuberosum*	5	Yunnan, China	+	+	+
*Alternaria solani*	*Solanum tuberosum*	6	Qujing, China	+	+	+
*Alternaria solani*	*Solanum tuberosum*	3	Zhaotong, China	+	+	+
*Alternaria solani*	*Solanum tuberosum*	4	Huize, China	+	+	+
*Alternaria solani*	*Solanum tuberosum*	1	Sanming, China	+	+	+
*Alternaria solani*	*Solanum tuberosum*	1	Longyan, China	+	+	+
*Alternaria solani*	*Solanum tuberosum*	8	Fujian, China	+	+	+
*Alternaria solani*	*Solanum tuberosum*	2	Fuzhou, China	+	+	+
*Alternaria solani*	*Solanum tuberosum*	3	Zhangzhou, China	+	+	+
*Alternaria alternata*	*Solanum tuberosum*	2	Fujian, China	-	-	N/A
*Alternaria alternata*	*Glycine max*	1	Fujian, China	-	-	N/A
*Alternaria citri*	*Citrus reticulata*	1	Fujian, China	-	-	+
*Alternaria raphani*	*Raphanus sativus*	1	CGMCC	-	-	-
*Alternaria tenuis*	*Apium graveolens*	1	CGMCC	-	-	N/A
*Alternaria mali*	*Malus pumila*	1	CGMCC	-	-	N/A
*Alternaria longipes*	*Nicotiana tabacum*	1	CGMCC	-	-	+
*Alternaria zinniae*	*Xanthium sibiricum*	1	CGMCC	-	-	-
*Alternaria porri*	*Allium cepa*	1	CGMCC	-	-	N/A
*Alternaria cucumerina*	*Cucumis sativus*	1	CGMCC	-	-	N/A
*Alternaria brassicae*	*Brassica campestris*	1	CGMCC	-	-	N/A
*Fusarium oxysporum*	*Solanum lycopersicum*	1	Fujian, China	-	-	+
*Rhizoctonia solani*	*Solanum tuberosum*	1	Fujian, China	-	_	+
*Botrytis cinerea*	*Vitis vinifera*	1	Fujian, China	-	-	-
*Colletotrichum gloeosporioides*	*Musa nana*	1	Fujian, China	-	-	-
*Sclerotinia sclerotiorum*	*Lactuca sativa*	1	Fujian, China	-	-	-
*Cercospora sojina*	*Glycine max*	1	Fujian, China	-	-	N/A
*Ceratocystis fimbriata*	*Dioscorea esculenta*	1	Fujian, China	-	-	N/A
*Botryosphaeria rhodina*	*Psidium guajava*	1	Fujian, China	-	-	N/A
*Pestalotiopsis pauciseta*	*Taxus chinensis*	1	Zhangping, China	-	-	N/A
*Aspergillus flavus*	*Arachis hypogaea*	1	Fujian, China	-	-	N/A
*Mycosphaerella melonis*	*Cucumis sativus*	1	Fujian, China	-	-	N/A
*Phytophthora infestans*	*Solanum tuberosum*	1	Fujian, China	-	-	N/A
*Phytophthora sojae*	*Glycine max*	1	Fujian, China	-	-	N/A
*Phytophthora capsici*	*Capsicum annuum*	1	Fujian, China	-	-	N/A
*Peronophythora litchi*	*Litchi chinensis*	1	Fujian, China	-	-	N/A
*Pythium aphanidermatum*	*Glycine max*	1	Fujian, China	-	-	N/A


*^*a*^All isolates of *Alternaria* spp. and fungal isolates were maintained in the collection of Fujian Academy of Agricultural Sciences. ^*b*^CGMCC for China General Microbiological Culture Collection. ^*c*^Note that presence (+) or absence (-) are based on the presence of a PCR or LAMP product of the expected size.*

### *A. solani* Primer Design

**FIGURE 1:**
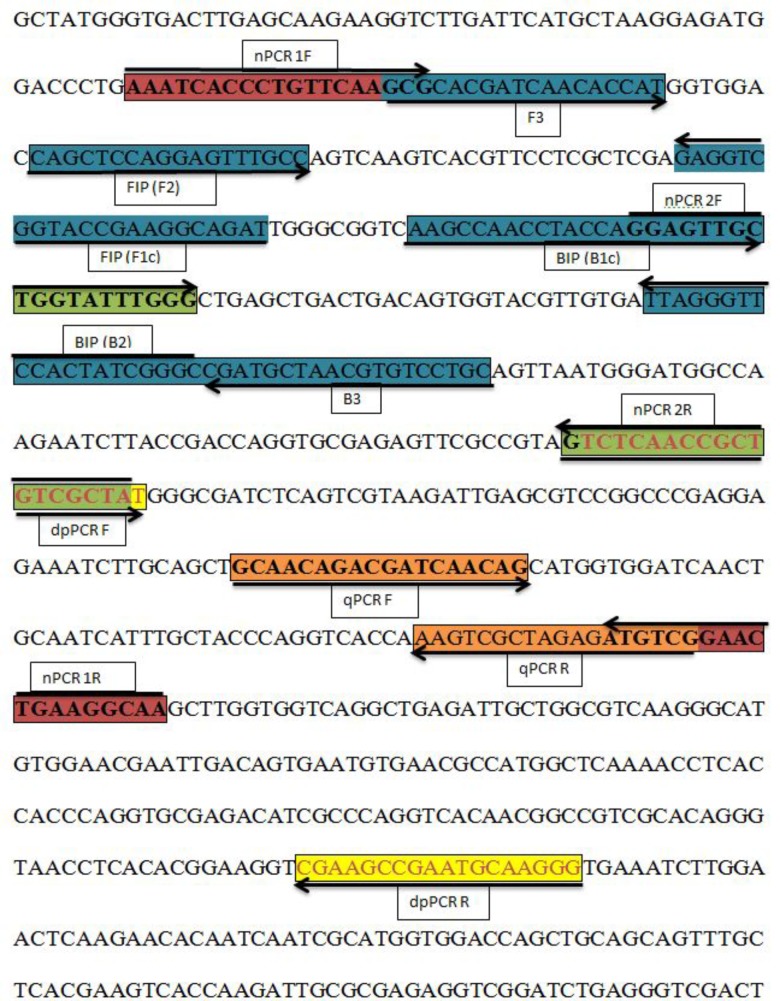
Sequence and location of the *A. solani histidine kinase* gene (HK1) used to design different polymerase chain reactions (PCRs) and loop-mediated isothermal amplification (LAMP) primers. Primer locations for different PCRs [Real-time PCR (qPCR F, qPCR R); Nested PCR (nPCR1 F, nPCR1 R, nPCR2 F, and nPCR2 R)], Duplex PCR (dpPCR F, dpPCR R), and LAMP assays [F3, B3, FIP (F1c-F2), BIP (B1c-B2)]. FIP is a hybrid primer consisting of the F1c and F2 sequences; BIP is a hybrid primer consisting of the B1c and B2 sequences. Primer sequence sites are colored and marked. Arrows indicate the extension direction.

In the present study, the *histidine kinase* gene (HK1) was targeted to amplify different isolates of *A. solani*, other *Alternaria* species, and other fungi to determine primer specificity. We selected the *histidine kinase* gene (Accession No. FJ424058.1), and pairs of primers were designed and cloned to confirm its efficiency. LAMP primers consisted of two outer primers (F3 and B3) and two inner primers (FIP and BIP) based on the *histidine kinase* gene (HK1) sequence and developed using the Primer Explorer V4 software^1^ (Eiken Chemical Co., Ltd., Tokyo, Japan). Duplex PCR primers and two pairs of nested PCR primers, each comprising outer and inner primers, were designed using the Primer Premier 5 software. The primers used for *Phytophthora infestans* in the duplex PCR assay were identified from a previous study (Khan et al., 2017). The nested PCR outer primers were also used in conventional PCR assays. The primers for the qPCR were designed using the Beacon software. Information on the locations and sequences of the primers used in this study is provided in **Table 2** and **Figure 1**.

**Table 2:** Primers used in this study.

Primer	Type Primer	Primer Sequences (5’ to 3’)
Nested PCR 1st round	nPCR 1F	AAATCACCCTGTTCAAGCG
	nPCR 1R	TTGCCTTCAGTTCCGACAT
Nested PCR 2nd round	nPCR 2F	GGAGTTGCTGGTATTTGGG
	nPCR 2R	TAGCGACAGCGGTTGAGAC
Real-time PCR	qPCR F	GCAACAGACGATCAACAG
	qPCR R	CGACATCTCTAGCGACTT
LAMP	F3	GCGCACGATCAACACCAT
	B3	GCAGGACACGTTAGCATCG
	FIP	ATCTGCCTTCGGTACCGACCTC-CAGCTCCAGGAGTTTGCC
	BIP	AAGCCAACCTACCAGGAGTTGC-GCCCGATAGTGGAACCCTAA
Duplex PCR for *A. solani*	dpPCR F	TCTCAACCGCTGTCGCTAT
	dpPCR R	CCCTTGCATTCGGCTTCG
Duplex PCR for *P. infestans*	Yph F	CGACCATKGGTGTGGACTTT
	Yph R	ACGTTCTCMCAGGCGTATCT


### Detection of *A. solani* by Conventional PCR

The reaction system consisted of 12.5 μL Taq PCR Master Mix (Tiangen, Beijing, China), 1 μL each of the forward and reverse primers (10 μM), 1 μL template DNA, and sterilized distilled water to a final volume of 25 μL. All conventional PCRs were performed in a PTC200 thermocycler (MJ Research, Watertown, MA, United States), with initial denaturation at 95°C for 3 min, followed by 26 cycles of denaturation at 95°C for 1 min, annealing at 58°C for 1 min, and extension at 72°C for 1 min, with final extension at 72°C for 5 min to evaluate the conventional PCR primers (CPCR-F/CPCR-R). Nested PCR comprised two rounds of amplification using conventional PCR primers in the first round; in the second round, the first-round product (about 1 μL) were used as template DNA by using nested primers. The PCR protocol was the same in both rounds, but with an annealing temperature of 64°C in the second round. Sensitivity and specificity tests were performed for the different PCR assays. The sensitivity of conventional and nested PCR was tested using 10-fold serial dilution of target genomic DNA with sterile double-distilled water from 136 ng to 136 fg, using the PCR programs mentioned above. All PCR experiments were repeated at least three times.

### LAMP Assay Optimization

The LAMP assay was optimized using different LAMP primer concentrations [F3/B3 (0.1–0.5 μM) and FIP/BIP (0.8–2.4 μM)], durations (30–70 min), temperatures (57–67°C), and reagents [dNTPs (0.8–2.0 mM), MgSO_4_ (0.5–3.0 mM), and betaine (1.5–4 M)]. The LAMP system used in this study consisted of 12.5 μL LAMP buffer, 4 μL primer mix (F3/B3 and FIP/BIP), 1 μL calcein-MnCl_2_, 1 μL template DNA, 1 μL *Bst* DNA polymerase (8000U, New England Biolabs), and sterile double-distilled water for a final volume of 25 μL. The LAMP reaction was conducted in a thermal water bath by adjustment to the optimized temperature of 63°C for 60 min. The amplified LAMP products were further observed on 2% stained agarose gel with ethidium bromide to confirm amplification. All reactions were repeated at least three times.

### Sensitivity of the LAMP Reaction

The sensitivity of the LAMP assay was determined using different *A. solani* DNA concentrations in descending order by 10-fold serial dilution with sterilized double-distilled water from 136 ng to 13.6 fg. Serially diluted DNA (1 μL each) was used as template DNA in the LAMP reaction to quantify its sensitivity in a thermal water bath at a uniform temperature of 63°C for 60 min. These reactions were repeated at least three times.

### LAMP Assay of Diluted Pure *A. solani* Culture

We performed LAMP assays using minute amounts of diluted pure culture from different isolates of *A. solani* as template DNA for the LAMP reaction system. *A. solani* isolates were grown on PDA medium at 28°C for 4–5 days, after which mycelia were diluted in sterile double-distilled water in 50-mL Falcon tubes. Spore concentrations were 7 spores/μL. We used 1 μL diluted pure culture as a template for the LAMP reaction, and then analyzed the product by gel electrophoresis and ethidium bromide staining. These experiments were repeated at least three times.

### LAMP Detection of Infected Field Samples

Infected and uninfected leaves were collected from different fields in the Zhouning and Ningde regions in China. Total DNA was extracted by the CTAB method and the NaOH rapid extraction method (Tooley et al., 1997; Zhang et al., 2006). A 10-mg sample of infected leaves was crushed using a glass pestle with 0.5 M NaOH, and centrifuged at 12,000 rpm for 5 min. The liquid phase was diluted immediately with 195 μL 100 mM Tris. The products were then used as a template for LAMP reactions, which were repeated at least three times.

### Duplex PCR Using Different Primers

Duplex PCR is a variant of PCR that enables simultaneous amplification of two target DNAs and their primer pairs. In this study, we performed duplex PCR using template DNA from *A. solani* and *P. infestans* and their specific primer pairs. The reaction system comprised 12.5 μL Taq PCR Master Mix (Tiangen), 1 uL each of *A. solani* and *P. infestans* forward and reverse primers, 1 μL template DNA from *A. solani* and *P. infestans*, and sterilized double-distilled water for a final volume of 26 μL. The duplex PCR reactions were performed in a PTC200 thermocycler (MJ Research) with initial denaturation at 95°C for 3 min, followed by 26 denaturation cycles at 95°C for 1 min, annealing at 60°C for 1 min, and extension at 72°C for 1 min, with final extension at 72°C for 5 min to amplify the template DNA. We used two positive controls (each with a primer pair and *A. solani* or *P. infestans* DNA) and one negative control lacking template DNA. The duplex PCR reactions were repeated at least three times.

### Sensitivity and Specificity Detection by Real Time-qPCR

Real time-qPCR reactions were performed in a CFX96 real-time detection system (Bio-Rad, Hercules, CA, United States) by adding Maxima SYBR Green I qPCR Master Mix (TaKaRa, Dalian, China) in 0.5-mL thin-walled, optical-grade PCR tubes. The reaction mixture comprised Maxima SYBR Green I qPCR Master Mix (12.5 μL), 1 μL 10 mM of each primer (qPCR F and qPCR R), target genomic DNA (1 μL), and sterilized double-distilled water for a final volume of 25 μL. The amplification conditions for the reaction were 95°C for 30 s, followed by 39 cycles at 95°C for 5 s, 60°C for 30 s, and 72°C for 20 s, with a fluorescence read at 72°C at the end of each cycle, and a final melting curve at 65–95°C at increments of 0.1°C s^-1^. The target genomic DNA was 10-fold serially diluted using Easy dilution liquid (TaKaRa) with an initial concentration of 1.36 × 10^2^ ng mL^-1^, diluted in a 1:10 sequence to 1.36 × 10^-4^ ng mL^-1^ for sensitivity check. For specificity check, three different isolates of *A. solani*, along with four different species (*Alternaria citri*, *Alternaria raphani*, *Alternaria longipes*, and *Alternaria zinniae*), infected field samples from four different fields and four other oomycetes fungi (*Rhizoctonia solani*, *Botrytis cinerea*, *Colletotrichum gloeosporioides*, and *Sclerotinia sclerotiorum*) were examined. C_t_ values of less than 35 were considered to be positive. The results were analyzed by calculating the log of the target DNA concentration against the cycle threshold (C_t_) scores, using the formula E = [10 (–1/slope)–1] × 100 to determine the PCR efficiency for determination of sensitivity. The reactions were repeated at least three times.

## Results

### Optimization of the LAMP Assay

We performed the LAMP assay with different LAMP reagent concentrations, durations, and temperatures to determine the optimized reaction system for the detection of *A. solani* genomic DNA by targeting the *histidine kinase* gene (HK1). The best reaction temperature and time found for *A. solani* target DNA were 63°C and 60 min, respectively. The best concentration for FIP/BIP was 1.2 μM, followed by F3/B3 at 0.4 μM, MgSO_4_ at 6 mM, dNTPs at 1.2 mM, and betaine at 0.6 M. Positive reactions yielded green color due to the quenching effect of calcein-MnCl_2_ fluorescent dye (Niu et al., 2012), and negative reactions yielded brown color (**Supplementary Figure S1**). The reaction products were then analyzed on 2% agarose gel stained with 1 μL ethidium bromide; ladder-shaped bands demonstrated the effectiveness of the LAMP primers.

### Specificity of *A. solani* Detection by LAMP and PCR Primers

**FIGURE 2:**
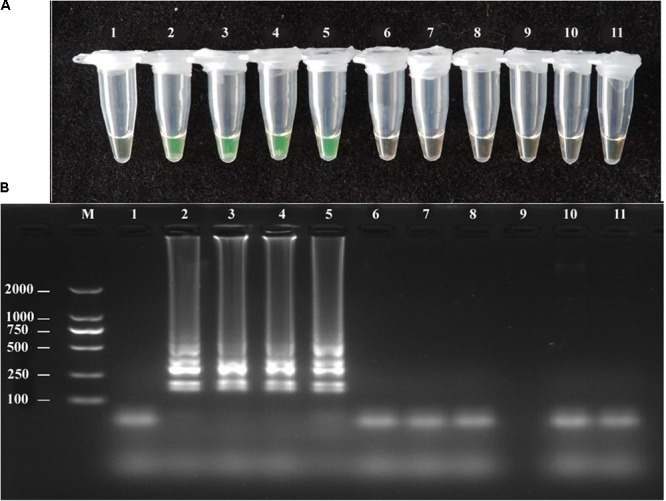
Specificity of LAMP detection of *A. solani*. Assessment was based on **(A)** Calcein visualization of color change and **(B)** agarose gel electrophoresis analysis of the LAMP products. Lane 1: negative control; Lane 2–5: *A. solani* from different geographic areas; Lane 6: *A. longipes*; Lane 7: *A. zinniae*; Lane 8: *A. porri*; Lane 9: *Colletotrichum gloeosporioides*; Lane 10: *Fusarium oxysporum*; Lane 11: *Pythium aphanidermatum*; Lane M: DL2000 DNA markers. Similar results were observed in three repeat assessments.

The *A. solani* isolates used in this study showed positive reactions in the LAMP assay, whereas other *Alternaria* species showed negative reactions (**Figure 2**). The LAMP and conventional PCR assays showed the same specificity (**Table 1**).

### Duplex PCR Specificity

Duplex PCR was assessed using *A. solani* and *P. infestans* in the same reaction tube as their primers. The duplex PCR products were 334 bp for *A. solani* and 497 bp for *P. infestans*, along with one positive control for each species and a negative control sterilized with sterilized, double-distilled water instead of template DNA (**Supplementary Figure S2**).

### LAMP and PCR Assay Sensitivity

**FIGURE 3:**
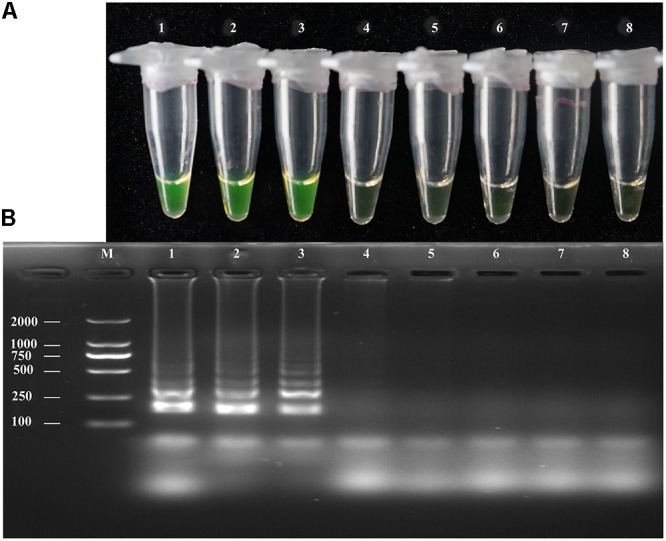
Sensitivity of the LAMP assays. LAMP assay using 10-fold serial dilutions of purified target DNA from *A. solani*. **(A)** Detection of LAMP products by Calcein fluorescence dye. **(B)** Analysis of the LAMP products by agarose gel electrophoresis. Concentrations of template DNA were as follows: Lane 1: 1.36 × 10^2^ ng μL^-1^; Lane 2: 1.36 × 10^1^ ng μL^-1^; Lane 3: 1.36 ng μL^-1^; Lane 4: 1.36 × 10^-1^ ng μL^-1^; Lane 5: 1.36 × 10^-2^ ng μL^-1^; Lane 6: 1.36 × 10^-3^ ng μL^-1^; Lane 7: 1.36 × 10^-4^ ng μL^-1^; Lane 8: negative control; Lane M: 2000-bp DNA marker. Similar results were observed in three repeat assessments.

The sensitivity of the LAMP, conventional PCR, and nested PCR assays was assessed by 10-fold serial dilution of *A. solani* genomic DNA. By naked eye, the LAMP assay showed sensitivity ranging from 1.36 × 10^2^ to 1.36 ng/μL^-1^; the LAMP reaction products were then subjected to 2% gel electrophoresis to confirm the amplifications (**Figure 3**). The sensitivity of conventional PCR ranged from 1.36 × 10^2^ to 1.36 × 10^-1^ ng/uL^-1^ (**Supplementary Figure S3**). The sensitivity of nested PCR was 1.36 × 10^-1^ ng/uL^-1^ (**Table 3** and **Supplementary Figure S4**).

**Table 3:** Comparison of the sensitivity of various PCR assays and LAMP conducted in this study.

	1.36 × 10^2^ ng μL^-1^	1.36 × 10^1^ ng μL^-1^	1.36 ng μL^-1^	1.36 × 10^-1^ ng μL^-1^	1.36 × 10^-2^ ng μL^-1^	1.36 × 10^-3^ ng μL^-1^	1.36 × 10^-4^ ng μL^-1^	1.36 × 10^-5^ ng μL^-1^
PCR	**+**	**+**	**-**	**-**	**-**	**-**	**-**	-
LAMP	**+**	**+**	**+**	**-**	**-**	**-**	**-**	-
Nested PCR	**+**	**+**	**+**	**+**	**+**	**-**	**-**	-
Real-time PCR	**+**	**+**	**+**	**+**	**+**	**+**	**-**	-


### LAMP Assay of Diluted Pure *A. solani* Culture

**FIGURE 4:**
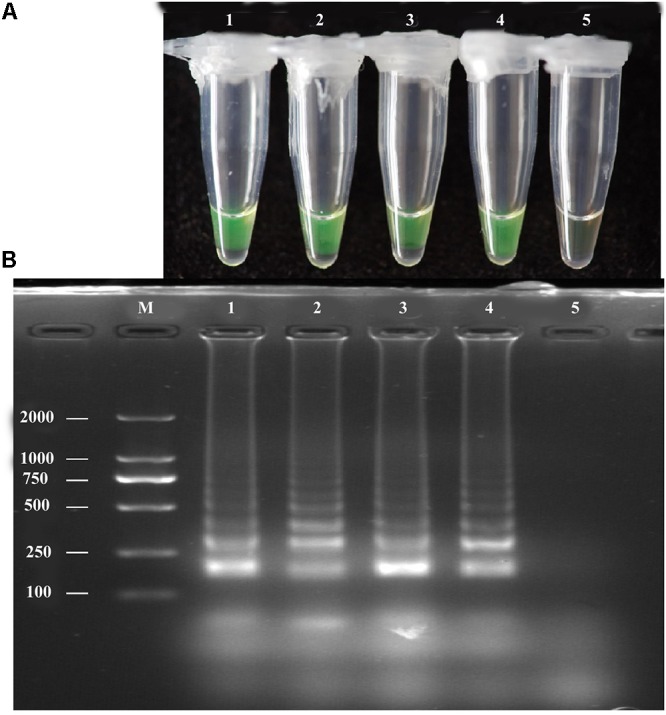
LAMP assay tested for diluted Pure cultures. **(A)**Visual inspection of three different diluted pure cultures of *A. solani* was tested for LAMP assay without DNA extraction. **(B)** Analysis of the LAMP products by 2% agarose gel electrophoresis. Lane M: DL2000-bp DNA marker; Lane 1–3: different *A. solani* isolates from different geographic areas; Lane 4: positive control (*A. solani* genomic DNA); Lane 5: negative control. Similar results were observed in three repeat assessments.

The LAMP assay was tested using a diluted pure *A. solani* culture grown on PDA medium. The spore concentration was about 7 spores/μL; 1 μL diluted pure culture was used as the target template. Positive amplification was recorded for diluted pure culture of different isolates and *A. solani* genomic DNA (used as a positive control), whereas a negative control containing sterile double-distilled water instead of DNA showed no amplification (**Figure 4**). The LAMP products were then analyzed by 2% gel electrophoresis and staining with ethidium bromide.

### Detection by Real Time-qPCR

**FIGURE 5:**
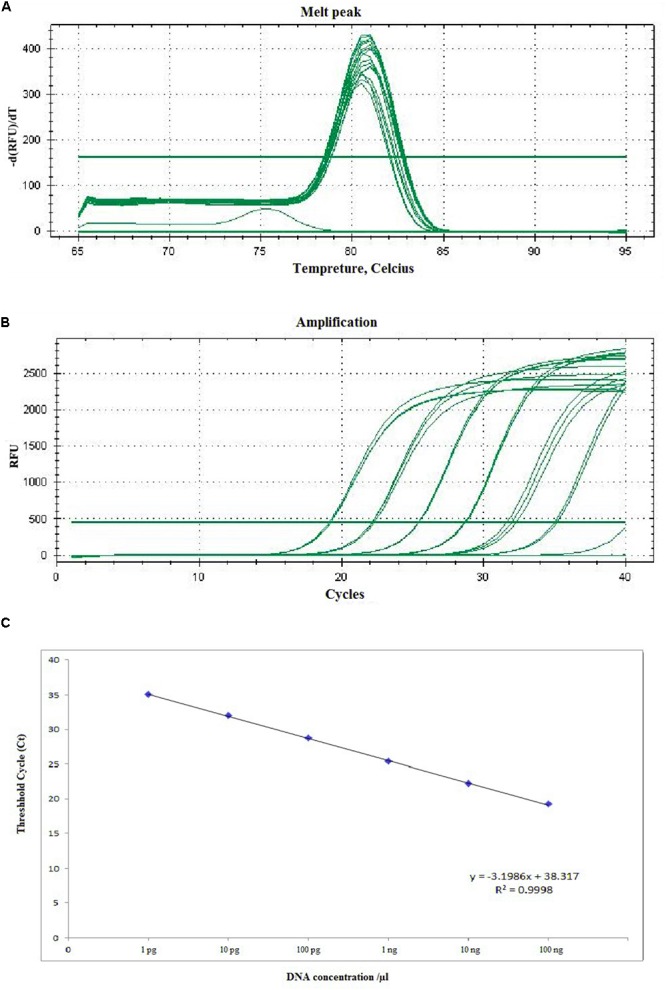
Representative melting curves **(A)**, amplification plot **(B)**, and standard curve **(C)** of real-time PCR for detection of *A. solani* based on the *histidine kinase* gene (HK1). **(A)** Demonstrative melting curves using SYBR Green I for detection of *A. solani*. **(B)** A representative amplification plot for 10-fold serial dilution containing 100 ng to 1 pg of genomic DNA. **(C)** Standard curve derived from absolute quantification of 10-fold serially diluted DNA from a pure culture of *A. solani*.

The sensitivity of RT-qPCR was determined using 10-fold serial dilution of *A. solani* DNA with Easy Dilution (TaKaRa). The C_t_ values ranged from 19.23 ± 0.058 to 35.19 ± 0.107. The sensitivity of RT-qPCR ranged from 1.36 × 10^2^ to 1.36 × 10^-3^ ng mL^-1^, amplifying the product to 77 bp (**Figure 5**). The C_t_ values and genomic DNA were strongly correlated (*y* = -3.1986*x* + 38.317, *R*^2^ = 0.9998). In case of specificity, the different isolates, species (*Alternaria citri* and *Alternaria longipes*), infected field samples, along with some other fungi (*Rhizoctonia solani*) showed positive results, while healthy plant DNA and negative control showed no amplification (**Supplementary Figure S5**).

### LAMP Detection of Infected Field Samples

**FIGURE 6:**
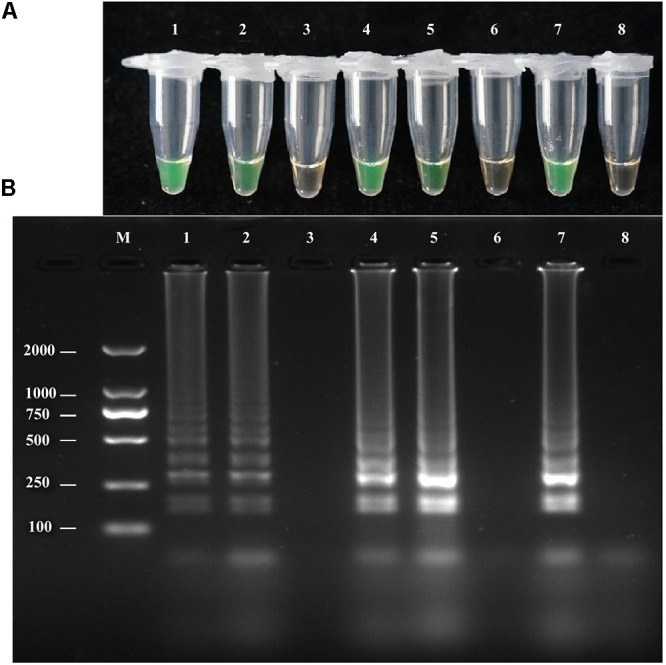
LAMP detection of *A. solani* from infected potato field samples. **(A)**Visual inspection of LAMP assay using two different DNA extraction methods (rapid DNA extraction and CTAB DNA extraction) from potato plant tissues (leaf and stem). **(B)** Analysis of the LAMP products by 2% agarose gel electrophoresis. Lane 1: DNA extracted by rapid extraction from infected potato leaf; Lane 2: DNA extracted from infected stem by rapid DNA extraction; Lane 3: healthy plant tissue DNA extraction by rapid DNA extraction; Lane 4: DNA extracted from infected leaf by the CTAB method; Lane 5: DNA extracted from infected stem by the CTAB method; Lane 6: DNA extracted from healthy plant tissues by the CTAB method; Lane 7: positive control; Lane 8: negative control; Lane M: DL2000-bp DNA marker. Similar results were observed in three repeat assessments.

To evaluate the performance of the LAMP assay with infected field samples, DNA was extracted from diseased and uninfected plants by two different extraction methods, as described above, and used as template target DNA in the LAMP reaction. Positive amplification detected infected samples and positive controls used in the reaction, whereas the negative control and healthy plant DNA did not exhibit positive amplification. Both extraction methods yielded the same results (**Figure 6**). The LAMP assay products were further analyzed by 2% gel electrophoresis with ethidium bromide staining.

## Discussion

To our knowledge, this study is the first to report on comparison of different PCR-based assays and the LAMP technique for the detection of *A. solani* targeting the *histidine kinase* gene (HK1). Post-amplification techniques, e.g., gel electrophoresis and large-scale application of conventional PCR, are laborious and time consuming for routine fungal pathogen diagnosis. Pathogen detection, identification, and quantification are important in plant disease control, and must be accessible in all regions to ensure sustainable crop production and food safety.

Real time-qPCR–based techniques have contributed greatly to plant disease management, emerging as robust tools for the diagnosis and quantification of *A. solani* in tomato seed-borne pathogens, thereby providing several benefits over conventional PCR-based techniques for plant disease detection, including increased sensitivity and no requirement for gel electrophoresis to quantify target DNA (Guillemette et al., 2004; Schena et al., 2004; Alaei et al., 2009; Debode et al., 2009).

In recent studies, the lower detection limits attained by real time-qPCR have made this technique attractive in the detection of *A. solani* by targeting the *β-tubulin* gene (Kumar et al., 2013). The *histidine kinase* gene (HK1) showed high sensitivity and specificity in the LAMP assay and qPCR assay (1.36 ng and 1.36 pg) in this study, unlike ITS region (10 ng for conventional PCR and 10 pg for real time PCR) (Chowdappa et al., 2014), which was used to detect *A. solani* in a previous study and yielded similar results to our conventional PCR assay results. A recent study about sensitive and rapid PCR assay, based on ITS regions of the ribosomal DNA, was developed to diagnose *A. brassicicola* or *A. japonica* infection of cruciferous seed (Iacomi-Vasilescu et al., 2002). But unfortunately, this method did not generate a reliable diagnosis when seeds were contaminated with *A. brassicae* because of cross-reactivity with other fungal species. In a more recent studies, Kumar et al., 2013 conducted experiment on real time quantitative analysis of *A. solani* resulting in 0.5 pg sensitivity targeting β-tubulin gene, which is 10 times more sensitive than the method developed in our study, which, however, provided feasibility of LAMP assay in the field. *A. solani* is an economically significant seed-borne pathogenic fungus that causes EB in potato and tomato. The production and supply of disease-free seeds can limit the production of this pathogen. Culture and morphology-based diagnostic approaches are expensive in terms of cost and effort. Real time-qPCR assays have been used to identify seed-borne fungi in previous studies, revealing that it can be a beneficial technique in seed health testing and quarantine inspections, due to its high specificity and sensitivity (Guillemette et al., 2004; Lievens et al., 2006; Alaei et al., 2009; Debode et al., 2009) but it did not show promising specificity in contrast to our findings. The current and previous studies have demonstrated that this method can be used for pathogen diagnosis in quarantine laboratories, disease screening programs, epidemiological studies, and fungicide resistance screening, but it did not provide on-site detection as LAMP assay does. A previous study showed that semi-nested PCR detected *A. solani* based on its region-specific primers (Gu et al., 2017). However, real time-qPCR cannot provide on-site diagnosis of the disease due to the need for expensive instruments and expert technicians. There are similarities between this study and some previous studies which demonstrate that LAMP assay has proven to be broadly functional in plant pathogen recognition (Tomlinson et al., 2010; Ash et al., 2014; Lu et al., 2015), due to its rapidity, sensitivity and specificity, as well as its adaptability to varied detection approaches and settings (Hodgetts et al., 2015; Jun-hai et al., 2015). Other studies have shown that the LAMP assay is a good substitute to conventional PCR-based methods for its rapidity, sensitivity, and uniform temperature requirements, making it more suitable than conventional PCR and other PCR strategies (nested PCR and real time qPCR), thereby providing on-site detection of a pathogen without requiring sophisticated equipments (Khan et al., 2017). Additionally, the LAMP reaction can be assessed visually (Liu et al., 2017). LAMP technologies may be useful in airborne inoculum detection and quantification assays performed with a spore trap system, as described recently for grapevine powdery mildew (Thiessen et al., 2016) and rice blast pathogen (Villari et al., 2017). In our study, the assays other than conventional PCR (10 ng) were very sensitive and able to detect 1.36 pg, 13.6 pg and 1 ng genomic DNA of the target fungi in the case of real time-qPCR, nested PCR and LAMP assay, respectively. The LAMP assay was also highly specific when evaluated against template DNA from a range of closely related and unrelated fungal species in lining our results with a previous study (Kumar et al., 2013) compared to specificity of qPCR in recent studies, but in our study the qPCR shows cross reactivity with some other species (*Alternaria citri* and *Alternaria longipes*) and fungi (*Rhizoctonia solani*). Furthermore, in our study these assays did not amplify healthy tomato DNA, but was able to amplify DNA from naturally infected field samples and also minute amount of diluted pure cultures.

In our comparison of LAMP and PCR assays, RT-qPCR showed the greatest sensitivity, followed by nested PCR, the LAMP assay, and conventional PCR. The LAMP assay proved to be applicable to field detection, potentially eliminating the need for expensive thermal cyclers, gel electrophoresis, and time-consuming DNA extraction methods. The LAMP specificity test showed no amplification in healthy plant tissues, closely related species, or other fungi, demonstrating the feasibility of LAMP as a potential field assay.

## Author Contributions

QC, QW, and MK conceived and designed the experiments and analyzed the data. MK, RW, BL, and PL performed the experiments. MK and QC wrote the paper. All the authors reviewed the manuscript.

## Conflict of Interest Statement

The authors declare that the research was conducted in the absence of any commercial or financial relationships that could be construed as a potential conflict of interest. The reviewers BF and handling editor declared their shared affiliation at time of review.

## Footnotes

1 http://primerexplorer.jp/elamp4.0.0/index.html

## Supplementary Material

FIGURE S1 Detection of *A. solani* using the optimized LAMP system. LAMP assay and visual inspection using a fluorescent metal indicator (calcein) observed by the naked eye. **(**A) Positive reactions turned green in the presence of Calcein and negative became brown. (B) The LAMP assay products were evaluated using 2% agarose gel electrophoresis. The ladder-like bands indicate a positive reaction for *A. solani*. Lane M, DL2000 DNA markers, tube 1 represents a positive reaction; tube 2 represents the negative control. The same results were obtained in three repeat assessments.

FIGURE S2 Specificity of the duplex PCR. Duplex PCR was used using the DNA of both Late blight (497 bp) pathogen and early blight (384 bp) pathogen along with their respective PCR Primer sets. Lane M: DL2000bp Marker; Lane 1: *Phytophthora infestans* and *Alternaria solani*; Lane 2: *P. infestans*; Lane 3: *Alternaria solani*; Lane 4: Negative control. Similar results were observed in three repeat assessments.

FIGURE S3 Sensitivity of conventional PCR. Sensitivity test of conventional PCR confirmed by 2% agarose gel electrophoresis by 10-fold serial dilution of the target DNA. Lane 1: DL2000-bp DNA Marker; Lane 2: 1.36 × 102 ng μL^-1^: Lane 3: 1.36 × 101 ng μL^-1^; Lane 4: 1.36 ng μL^-1^; Lane 5: 1.36 × 10^-1^ ng μL^-1^; Lane 6: 1.36 × 10^-2^ ng μL^-1^; Lane 7: 1.36 × 10^-3^ ng μL^-1^; Lane 8: 1.36 × 10^-4^ ng μL^-1^; and Lane 9: negative control.

FIGURE S4 Sensitivity test of Nested PCR. Nested PCR sensitivity was tested using 10-fold serial dilutions of purified target DNA of *A. solani*. Analysis of Nested PCR products by Agarose gel electrophoresis. Concentrations of template DNA were as follows: Lane1: 1.36 × 102 ng μL^-1^; Lane 2: 1.36 × 101 ng μL^-1^; Lane 3: 1.36 ng μL^-1^; Lane 4: 1.36 × 10^-1^ ng μL^-1^; Lane 5: 1.36 × 10^-2^ ng μL^-1^; Lane 6: 1.36 × 10^-3^ ng μL^-1^; Lane 7: 1.36 × 10^-4^ ng μL^-1^; Lane 8: negative control; and Lane M: DL500-bp DNA marker. Similar results were observed in three repeat assessments.

FIGURE S5 Specificity of Real time-qPCR. 27 Amplification Curve: Specificity of real-time qPCR individual DNA templates, total DNA of *A. solani* isolates extracted from pure culture collected from different geographic areas, other species (*Alternaria citri*, *Alternaria raphani*, *Alternaria longipes*, and *Alternaria zinniae*), other fungal DNA ( *Rhizoctonia solani*, *Botrytis cinerea*, *Colletotrichum gloeosporioides*, and *Sclerotinia sclerotiorum* ), total DNA of infected field samples, and healthy plants (negative control) and sterile H2O (blank control). Three technical replicates were used in each DNA concentration.
