# Supplementary figures and images for: Comparative Evaluation of the LAMP Assay and PCR-Based Assays for the Rapid Detection of Alternaria solani
Source: Front Microbiol. 2018 Sep 3;9:2089. doi: 10.3389/fmicb.2018.02089 (PMC6129767; doi:10.3389/fmicb.2018.02089)

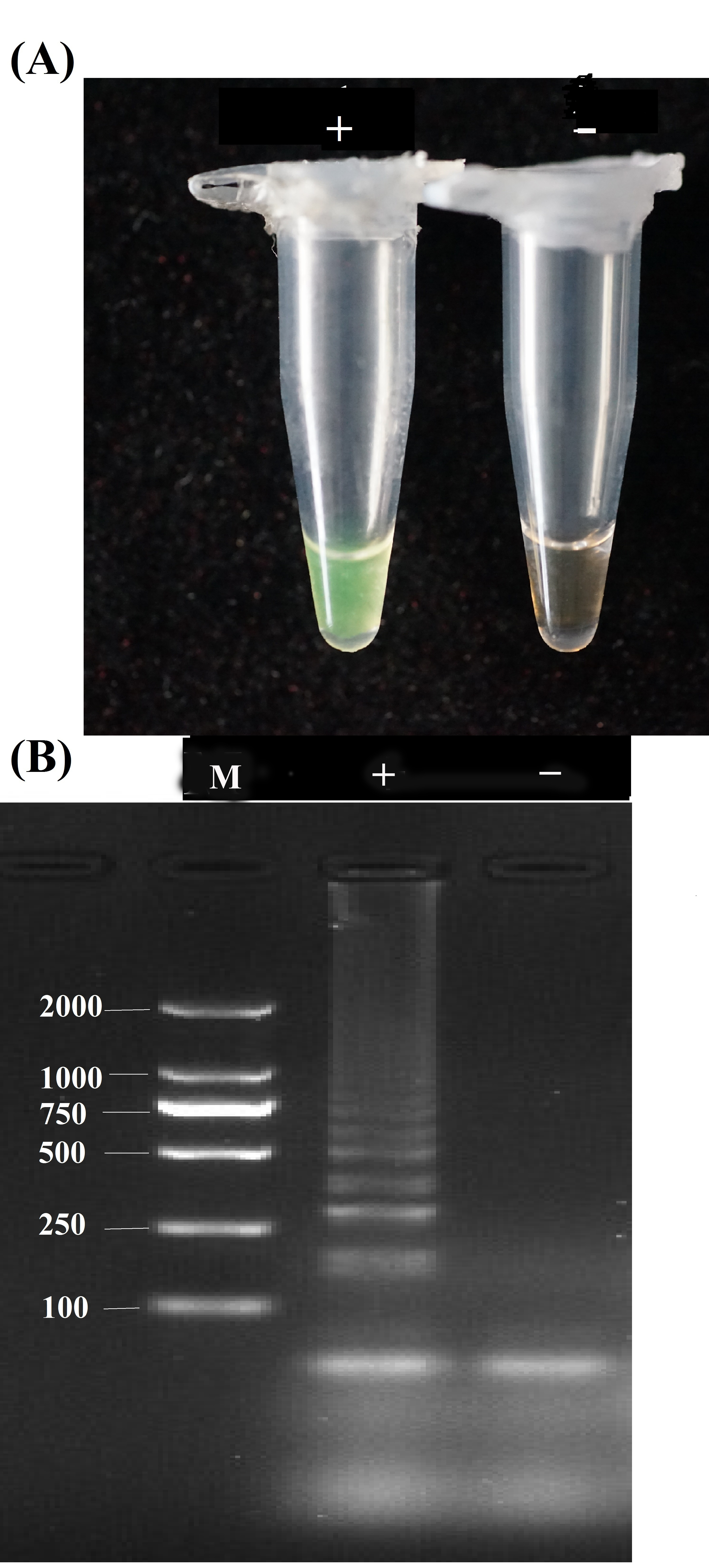

Supplement: FIGURE S1 — Detection of A. solani using the optimized LAMP system. LAMP assay and visual inspection using a fluorescent metal indicator (calcein) observed by the naked eye. (A) Positive reactions turned green in the presence of Calcein and negative became brown. (B) The LAMP assay products were evaluated using 2% agarose gel electrophoresis. The ladder-like bands indicate a positive reaction for A. solani. Lane M, DL2000 DNA markers, tube 1 represents a positive reaction; tube 2 represents the negative control. The same results were obtained in three repeat assessments. [file Image_1.JPEG]

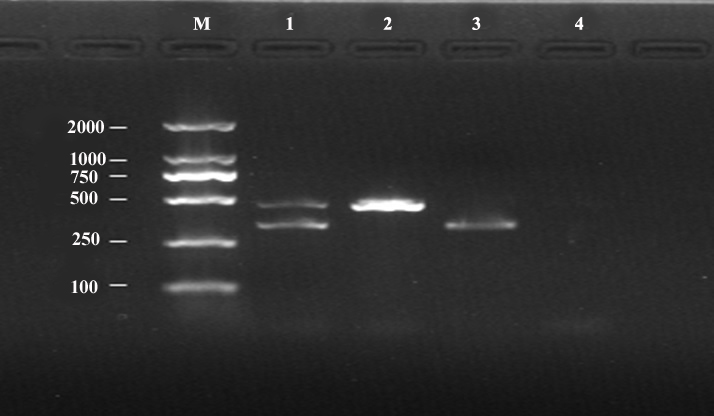

Supplement: FIGURE S2 — Specificity of the duplex PCR. Duplex PCR was used using the DNA of both Late blight (497 bp) pathogen and early blight (384 bp) pathogen along with their respective PCR Primer sets. Lane M: DL2000bp Marker; Lane 1: Phytophthora infestans and Alternaria solani; Lane 2: P. infestans; Lane 3: Alternaria solani; Lane 4: Negative control. Similar results were observed in three repeat assessments. [file Image_2.JPEG]

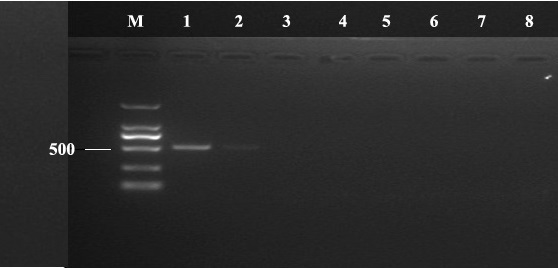

Supplement: FIGURE S3 — Sensitivity of conventional PCR. Sensitivity test of conventional PCR confirmed by 2% agarose gel electrophoresis by 10-fold serial dilution of the target DNA. Lane 1: DL2000-bp DNA Marker; Lane 2: 1.36 × 10^2^ ng μL-1: Lane 3: 1.36 × 101 ng μL-1; Lane 4: 1.36 ng μL-1; Lane 5: 1.36 × 10^-1^ ng μL-1; Lane 6: 1.36 × 10^-2^ ng μL-1; Lane 7: 1.36 × 10^-3^ ng μL-1; Lane 8: 1.36 × 10^-4^ ng μL-1; and Lane 9: negative control. [file Image_3.JPEG]

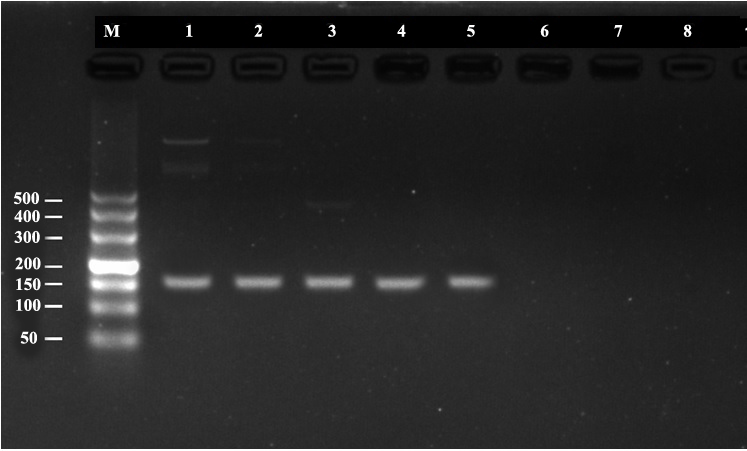

Supplement: FIGURE S4 — Sensitivity test of Nested PCR. Nested PCR sensitivity was tested using 10-fold serial dilutions of purified target DNA of A. solani. Analysis of Nested PCR products by Agarose gel electrophoresis. Concentrations of template DNA were as follows: Lane1: 1.36 × 10^2^ ng μL-1; Lane 2: 1.36 × 101 ng μL-1; Lane 3: 1.36 ng μL-1; Lane 4: 1.36 × 10^-1^ ng μL-1; Lane 5: 1.36 × 10^-2^ ng μL-1; Lane 6: 1.36 × 10^-3^ ng μL-1; Lane 7: 1.36 × 10^-4^ ng μL-1; Lane 8: negative control; and Lane M: DL500-bp DNA marker. Similar results were observed in three repeat assessments. [file Image_4.JPEG]

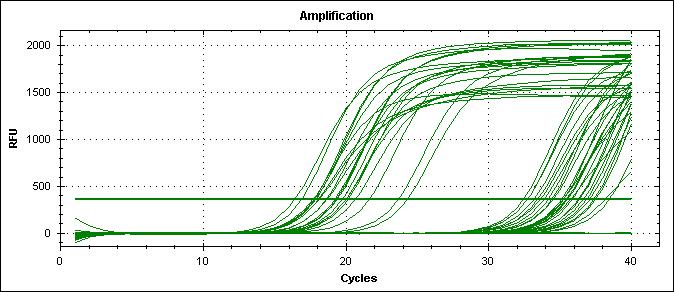

Supplement: FIGURE S5 — Specificity of Real time-qPCR. 27 Amplification Curve: Specificity of real-time qPCR individual DNA templates, total DNA of A. solani isolates extracted from pure culture collected from different geographic areas, other species (Alternaria citri, Alternaria raphani, Alternaria longipes, and Alternaria zinniae), other fungal DNA ( Rhizoctonia solani, Botrytis cinerea, Colletotrichum gloeosporioides, and Sclerotinia sclerotiorum ), total DNA of infected field samples, and healthy plants (negative control) and sterile H2O (blank control). Three technical replicates were used in each DNA concentration. [file Image_5.JPEG]
